# A longitudinal, multi-centre, superiority, randomized controlled trial of internet-based cognitive behavioural therapy (iCBT) versus treatment-as-usual (TAU) for negative experiences and posttraumatic stress following childbirth: the JUNO study protocol

**DOI:** 10.1186/s12884-018-1988-6

**Published:** 2018-10-01

**Authors:** Josefin Sjömark, Thomas Parling, Maria Jonsson, Margareta Larsson, Agneta Skoog Svanberg

**Affiliations:** 1 Department of Women’s and Children’s Health, Uppsala University, Akademiska sjukhuset, SE-751 85 Uppsala, Sweden; 2 Centre for Psychotherapy Education & Research, Stockholm Health Care Services, Stockholm County Council & Department of Clinical Neuroscience, Karolinska Institutet, Liljeholmstorget 7B, SE-113 64 Stockholm, Sweden

**Keywords:** Study protocol, iCBT, Immediate caesarean section, Negative birth experience, Postpartum haemorrhage, Posttraumatic stress following childbirth, PTSD following childbirth, PTSD

## Abstract

**Background:**

About one-third of women report their childbirth as traumatic and up to 10% have severe traumatic stress responses to birth. The prevalence of Posttraumatic stress disorder following childbirth (PTSD FC) is estimated to 3%. Women with PTSD FC report the same symptoms as other patients with PTSD following other types of trauma. The effect of psychological treatment for women with PTSD FC has only been studied in a few trials. Similarly, studies on treatment needs for women not diagnosed as having PTSD FC but who nevertheless face psychological problems are lacking.

**Methods/design:**

Women who rate their overall birth experience as negative on a Likert scale, and/or had an immediate caesarean section and/or a major postpartum haemorrhage are randomized to either internet delivered cognitive behaviour therapy (iCBT) plus treatment as usual (TAU) or TAU. The iCBT is to be delivered in two steps. The first step consists of six weekly modules for both the woman and her partner (if they wish to participate) with minimal therapeutic support. Step 2 consists of eight weekly modules with extended therapeutic support and will be offered to participants whom after step 1 report PTSD FC. Assessments will be made at baseline, 6 weeks, 14 weeks, and at follow-ups at 1, 2, 3 and 4 years after baseline. The primary outcome measures are symptoms of posttraumatic stress and depression. Secondary outcomes are quality of life, parent-child bonding, marital satisfaction, coping strategies, experience regarding the quality of care received, health-related quality of life, number of re-visits to the clinic and number of appointments for counselling during the 4 years’ period after the negative childbirth experience, time until the woman gets pregnant again, and the type of birth in the subsequent pregnancy. A health economic evaluation in the form of a cost utility analysis will be conducted.

**Discussion:**

This study protocol describes a randomized controlled trial that will provide information about the effectiveness of iCBT in women with negative experiences, posttraumatic stress, and PTSD FC.

**Trial registration:**

ISRCTN39318241. Date for registration 12/01/2017, retrospectively registered.

## Background

### Negative experiences following childbirth

Approximately 110,000 women give birth in Sweden annually [1]. Childbirth can be associated with both positive and negative psychological reactions, and a woman’s negative experience of labour and birth can affect her emotional wellbeing [2]. Between 9 and 45% of women reported a traumatic childbirth [3–5]. The experience of labour and birth is subjective, multidimensional and complex including physiological and psychological factors. There are several risk factors for negative childbirth-experiences such as unexpected medical problems, lack of social support, emotional state and care given during labour [2]. Negative and traumatic experiences during childbirth can sometimes lead to the development of posttraumatic stress symptoms and even posttraumatic stress disorder (PTSD).

### Posttraumatic stress following childbirth

In a meta-analysis, the prevalence for PTSD following childbirth (PTSD FC) was 3.1% among community samples and 15.7% among high-risk samples (e.g. pregnancy complications, emergency caesarean section, preterm birth) [6]. In a more recent review [7], the estimated prevalence was 2.4% among community samples, and 4.9% including both community plus high-risk samples when controlling for previous PTSD. The prevalence for clinically significant post-traumatic stress symptoms ranged between 9.6 and 27.3% [7]. The variation in prevalence rates may be explained by the timing and method used for assessment of PTSD criteria and symptoms [6, 7]. Prevalence rates were consistently higher in both studies when assessed close to childbirth. The prevalence rates above are based on the fourth edition of the Diagnostic and Statistical Manual for Mental Disorders (DSM-IV-TR) [8]. There is no sub-clinical or partial PTSD diagnose in DSM-IV, which is reflected in the heterogeneity regarding definition and label of clinically significant post-traumatic stress symptoms.

The definition of stressors for the development of PTSD according to DSM-IV can be applied to posttraumatic stress FC. A diagnosis of PTSD required affirming the following criteria: the stressor criterion (A) included stressful situations in which a person experience, witness, or is confronted with an event that involves actual death or threat of death or serious injury, or a threat to the physical integrity of self or others; the three symptom criteria re-experiencing (B), avoidance (C), and increased arousal (D); the duration criterion (E), and the influence criterion (F). Several risk factors are associated with PTSD FC, such as psychological factors (e.g. fear of childbirth), prepartum and partum factors (e.g. type of delivery) and postpartum factors (e.g. poor coping) [6, 9]. In a systematic review, five categories of risk factors for PTSD FC are identified: negative perception of childbirth, maternal mental health, history of trauma and PTSD, delivery mode and complications, and low social support [7].

Beside the symptoms of PTSD FC there is a strong association with postpartum depressive symptoms [6, 10] and comorbidity rates between 20 and 75% have been reported [10, 11]. Dysfunctional coping is common [12] and in terms of quality of life, women with PTSD FC reported negative changes in their mood, psychological well-being and social interaction [13]. PTSD FC and depression can also seriously affect the mother’s bonding with the baby with initial feelings of rejection towards the baby and in the long term she might develop either an avoidant or anxious attachment with her child [13]. Several aspects of infant development and behaviour, including patterns of eating, sleeping, and cognition, are mediated by parental posttraumatic reactions and depression [14]. Breastfeeding can also be negatively affected after a negative childbirth experience [15]. There is a higher risk of negative impact on the partner-relationship among women with PTSD FC than among women without. Sexual dysfunction and disagreements are common [13, 16] and those with PTSD FC and depression are more likely to avoid sexual relationships, wait longer before they get pregnant again, and give birth to fewer children [17, 18]. Few studies have examined the role of the partner and how his/her experience may affect the mother, the child, parenthood and any future plans to have more children [19]. It is suggested that providing additional support to partners in the early postpartum period may help alleviate any acute distress and reduce the detrimental impact the early postnatal symptoms may have on the partners [16]. Partner support seems important at all stages: stronger social partner support at mid-pregnancy was associated with lower emotional distress after childbirth [20] and stronger postnatal emotional partner support was associated with lower levels of symptoms of depression and hyper arousal [21].

### Interventions for women with negative experiences and PTSD FC

There has been relatively little research on psychological treatment for women with negative birth-experiences and PTSD FC [9]. The focus of this research has mostly been on postpartum counselling and debriefing (psychological interventions intended to reduce the psychological morbidity that arises after exposure to trauma). Results indicate that, in general, debriefing does not prevent psychiatric disorders or mitigate the effects of traumatic stress, even though people generally find debriefing helpful in the process of recovering from traumatic stress [22]. Gamble and Creedy [23] concluded that postpartum counselling and debriefing are often only general and nonspecific, and are not described in detail for replication. In addition, these interventions should be given by trained psychotherapists only. A systematic review [24] showed no preventive effect from a single debriefing session but did show some risk for developing PTSD. Postnatal debriefing should not be offered routinely after a traumatic birth [25].

The effects of structured writing assignments for psychological health have been investigated in many experiments [26] often using the intervention expressive writing developed by Pennebaker [27]. Structured writing about emotions associated with traumatic events have a positive effect on psychological wellbeing [28]. Expressive writing for new mothers reduced their symptoms of depression and posttraumatic stress [29]. Pre-term birth mothers were randomized to expressive writing or TAU and the intervention was acceptable and reduced post-traumatic stress, depression and improved mental health [30]. A pilot randomized controlled trial (RCT) investigated the effect of a visuo-spatial task (playing tetris) within 6 hours after emergency caesarean section compared with TAU [31]. The study showed significantly fewer intrusive memories in the intervention group compared with the control group 1 week after the intervention. This intervention was acceptable among a majority of participants and is interesting due to its simplicity and to the fact that it is easily administered.

The recommended treatment for PTSD is trauma-focused cognitive behaviour therapy (TFCBT) or eye movement desensitisation reprocessing (EMDR) [32]. TFCBT is the treatment that currently has the strongest research support for PTSD [33]. The theoretical framework for TFCBT involves confronting trauma related emotional distress via exposure that aim at disconfirming the beliefs that underlie and maintain the PTSD symptoms [34]. In particular, in vivo and imaginal exposure aim at disconfirming beliefs and perceptions related to traumatic stimuli (both external and internal). In vivo exposure enables new learning by activating distress and subsequent the experience of being capable of coping with the symptoms. Revisiting and recounting the trauma memory via imaginal exposure reorganizes the memory and generate habituation so that the trauma memory can be revisited without harm. It is suggested that the effect of TFCBT for those with PTSD FC is equivalent to those with PTSD due to other events [35]. There is no specific treatment recommendation for PTSD FC in Sweden but in the United Kingdom and some other western countries the treatment recommendations are based on existing knowledge about treatment of PTSD in general [36, 37].

To the best of our knowledge, no RCT with face to face CBT for women with PTSD FC has been published. A few case studies have investigated the effects of psychological interventions (CBT, EMDR and expressive writing), with positive results, for women with PTSD FC [15, 38–41]. However, one Swedish RCT investigated internet-delivered CBT (iCBT) vs waitlist for women with PTSD FC with recruitment taking place by using a nationwide invitation [42]. The iCBT (trauma focused) consisted of 8 weeks with support, which included feedback on homework assignments. The results (pre-post) showed a large between-group effect size at post (ES, *d* = 1.25) on the PTSD measure and a small but significant ES on depressive symptoms in favour of the iCBT group. The study was underpowered, but the authors conclude that iCBT can be used to help women with childbirth-related PTSD symptoms [42]. Depressive symptoms are common among mothers with or without PTSD or negative experiences. A recent trial investigated the effect of a brief online self-help (cognitive skills for negative beliefs) compared to an active comparison group (time management skills) [43]. There was a significant improvement in mood for the cognitive skills group compared to the control group. The authors conclude that online interventions for negative mood is acceptable and helpful for those without a clinical depression.

In a meta-analysis on iCBT for PTSD due to other events than negative effects of childbirth, medium to large effect sizes were found (0.66 < *g* < 0.83) in favour of iCBT vs passive controls [44]. The authors conclude that iCBT for PTSD is promising and a viable approach for treatment of PTSD. However, more studies with follow-up are needed [44], and the efficacy of iCBT for those with a negative experience needs to be evaluated [9]. Finally, we found no CBT or EMDR study that involved the partners of women with negative experiences or PTSD FC. There is also a need for health economic evaluations when investigating treatment interventions; none of the above-mentioned treatment studies presented this.

The current study addresses gaps concerning treatment for this population. First, the intervention consists of two steps, where step-1 is offered to all women with negative birth experiences, regardless of whether they have a PTSD diagnose or not. Those who fulfil the criteria for PTSD FC after step-1 are offered step-2. Second, the partners are invited to join the treatment in step-1. We also include long-term follow ups. The study involves the partner by facilitating a shared treatment experience with the mother, giving information on how to support her emotionally, and providing practical support that may help her to get the greatest benefit from the treatment program. Finally, we will present a health economic evaluation.

### Aim

The aims of this study are to investigate the effectiveness and cost-effectiveness of iCBT compared with TAU in women with negative birth experiences, posttraumatic stress and PTSD FC, and to investigate whether partner support may add a beneficial effect. The SPIRIT guideline was used.

## Method

### Design

**Fig. 1:**
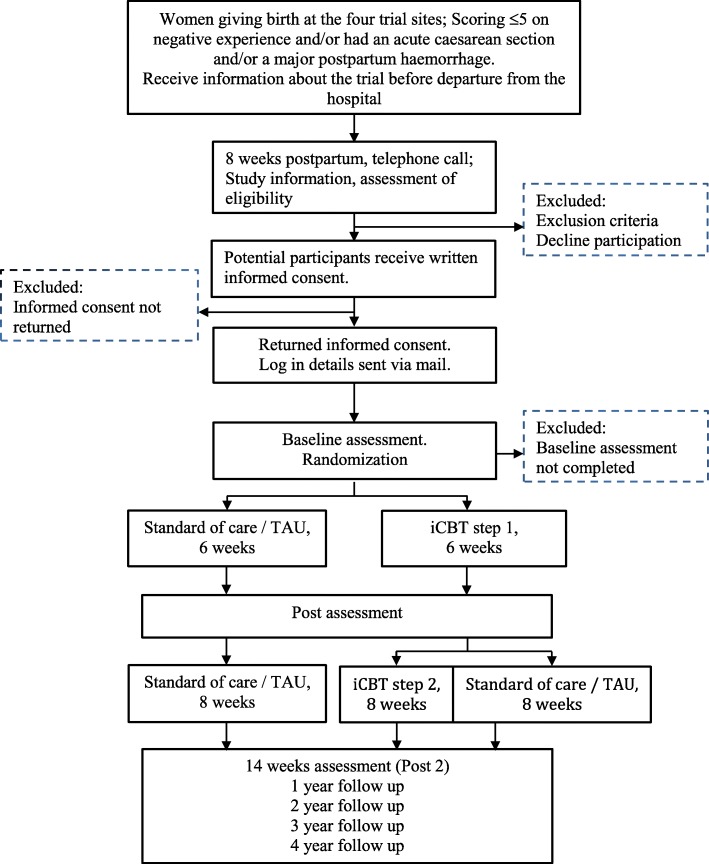
JUNO flow diagram

The study is a longitudinal, multi-centre, superiority, randomized controlled trial with two study arms; iCBT+TAU vs. TAU. Primary and secondary outcomes are assessed at baseline, after 6 and 14 weeks and subsequently at 1, 2, 3 and 4 years after end of treatment (Fig. 1).

### Hypothesis

#### Primary

Women who report a negative experience from childbirth and receive iCBT+TAU will report lower levels of posttraumatic stress and depressive symptoms, as well as a smaller proportion of participants classified as fulfilling the criteria for PTSD, and a smaller proportion of participants with postpartum depression according to cut-off compared with those receiving only TAU.

#### Secondary

Women who receive iCBT +TAU compared to those receiving only TAU will

- report a higher quality of life, better coping strategies, a more functional parent-child bonding, and a higher marital/partner satisfaction.
- have fewer re-visits to the clinic and counselling appointments.
- become pregnant and have vaginal births to a greater extent than the women receiving only TAU.

In addition, during the study period, a health economic evaluation will be performed and the preliminary effects of partner support will be investigated.

### Participants

Participants will be recruited at four hospitals in Sweden: Uppsala University hospital with 4000 annual births; Örebro University hospital with 2700 annual births; Linköping University with 2800 annual births, and Falun regional hospital with 3000 annual births.

Women, aged ≥18 years with a negative birth experience and/or an immediate caesarean section and/or a major postpartum haemorrhage are eligible. Exclusion criteria are severe mental illness, stillbirth, neonatal death, ongoing CBT treatment, difficulties understanding Swedish and women who are unable to use the Internet on a computer.

### Sample size and power

There was a lack of studies for power calculation regarding iCBT for this population at the planning stage of this study. We therefore used information from a study that investigated iCBT for PTSD (due to other events) in a Swedish context [45]. This study had a large effect size on the continuous primary outcome measure (*d* = 1.25) in favour of the treatment group. We estimated a conservative medium effect size (Cohen’s *d* = 0.5), *α* = .05, and a power of 0.8 based on this study. A total sample size of 130 participants is needed for between-subjects t-test contrasts. For categorical outcome measures, a medium effect size (*ω* = 0.3), *α* = .05, df = 1, and a power of 0.8 was used to calculate the needed sample size. A total sample size of 88 participants is needed for *χ*^2^ analysis.

### Procedure

The Care Base Internet Platform including its web-based part (U-CARE eService) was developed within the U-CARE program at Uppsala University. The aim of the U-CARE research program is to prevent and reduce psychosocial malfunctioning in patients and relatives. The U-CARE eService is currently being used for interventions and data collection http://www.u-care.uu.se. The present study is associated with the U-CARE program/eService.

**Fig. 2:**
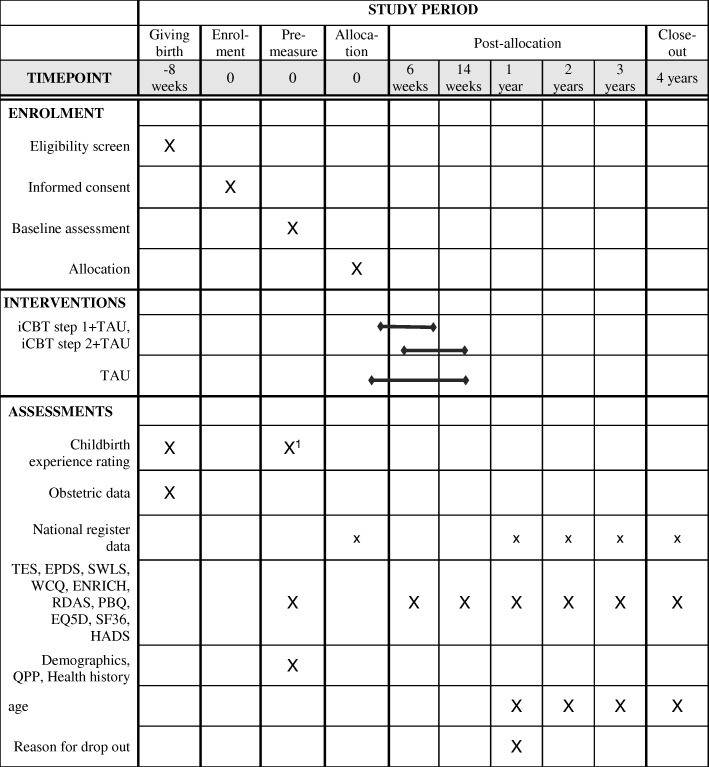
JUNO flow diagram. Note. *TAU* treatment as usual; iCBT=internet delivered cognitive behavior therapy *TES* The Traumatic Event Scale, *EPDS* Edinburgh Postnatal Depression Scale, *SWLS* The Satisfaction with Life Scale, *WCQ* The Ways of coping questionnaire, *ENRICH* The communication subscale from the Evaluation and Nurturing Relationship Issues, Communication and Happiness, *RDAS* The Revised Dyadic Adjustment Scale, *PBQ* The Postpartum Bonding Questionnaire, *EQ-5D* EuroQol 5D, *SF-36* Short Form Health Survey – 36, *HADS* Hospital Anxiety and Depression Scale, National register data = The medical birth register, the national patient register, the Swedish prescribed drug register, *QPP* Quality from the patient’s perspective, Health history = Information about previous pregnancies and births are collected at baseline; Obstetric data regarding pregnancy = childbirth, the post partum period and other medical data concerning the current childbirth X^1^ approximately 6-8 weeks after giving birth, this includes assessment of depression (EPDS)

Before discharge from hospital, women rate their overall childbirth experience on a paper and pencil administered Likert-scale (range; 0–10), please see Figure 2. Those with a low rating on the childbirth experience, defined as ≤5 on the Likert scale, and/or exposure to an immediate caesarean section and/or a major bleeding (≥2000 ml) following childbirth (see material section) are asked to consider participation in the study. Immediate caesarean section, is defined as when used for life-threatening situations for either the baby or the mother. About 8 weeks postpartum the women are contacted and informed about the study via telephone. To those interested in participation, information and consent is sent home by mail and to those not responding to contact after three attempts. Partners are also invited to participate at the same time as the mother and they sign the same consent form after reading the information about the study. Participants who consent are provided with login details to the Internet study site. The login and reporting procedures are as follows: Each login requires double authentication. At the first login, participants respond to baseline measures after which they are randomly allocated to either TAU or iCBT+TAU. They are immediately informed about the allocation via an email from the web portal. The partners are allocated together with the mothers. iCBT-1 + TAU and TAU are initiated immediately after randomisation. Participants respond to the questionnaires after iCBT step-1 (at 6 weeks) and after iCBT step-2 (at 14 weeks) followed by measurements annually up to 4 years post treatment. The U-CARE platform automatically sends reminders via email and text messages when measurements are not completed within 14 days. After another 7 days, participants are reminded via a telephone call. Paper copies of the questionnaires will be sent by mail to unresponsive participants. Reminders are also sent to those who delay their initiation of iCBT treatment and when participants delay the weekly treatment modules.

### Randomisation

After completion of the baseline assessment participants are allocated on a 1:1 ratio to either iCBT+TAU or TAU via the U-CARE system. Until randomisation is completed, the researcher is blind regarding the baseline questionnaire-answers. The participants’ allocation is not concealed for any additional caregiver involved and may be disclosed by the participants if they so choose.

### Interventions

#### iCBT step-1 + TAU

The first step of the intervention encompasses iCBT with therapist support at request. Participants can read the material and/or choose to listen to an audio-visual presentation as well as viewing videos covering e.g. breathing retraining and vignettes of childbirth stories. It also includes psychoeducation and basic interventions and techniques that aim to improve health and alleviate symptoms caused by posttraumatic stress and depression [46] (Table 1). The six weekly modules include homework based on the content. The partner receives identical content and a ‘partner instruction’ with specific tasks to perform (support and make room for practice; reflective listening; talk about the birth experience and present symptoms etc.) A psychologist guide, encourage, and respond to questions from the participants. After the completion of each module the psychologist writes an email to the participant to inform her that the week’s CBT is completed. Participants will be informed on a regular basis that they may ask questions or consult the psychologist via email.

**Table 1:** Overview of the of the iCBT step-1 week by week for the woman and her partner

	Woman	Partner
Week 1	Information, psychoeducation, breathing retraining	Take part of the material, facilitate, make time, homework reminder
Week 2	Vignettes, common symptoms, fear and avoidance	Discuss the information, remind and encourage practicing
Week 3	Depressive symptoms, significance of relations,“ reflective listening”	Reflective listening, encourage talk about childbirth, remind/encourage practice
Week 4	Exposure, talking about the childbirth	Watch pictures & movies, talk about the experiences, support practice
Week 5	Managing anxiety and depressive symptoms, psychological health, values, recovery	Talk about values, psychological health, and relaxing high demands
Week 6	Summary, repetition and relapse prevention	Talk about the information, make time to summarize, maintenance plan

Note. The content is identical for the partner and the mother except that the partner has additional information with instructions on what to do, discuss, facilitate etc

^1^Every week contains homework assignments based on the content of the module

The women who complete iCBT step-1 and fulfil the criteria for PTSD (according to The Traumatic Event Scale) will be offered iCBT step-2.

#### iCBT step-2 + TAU

The psychological treatment in iCBT step-2 is structured and follows an evidence-based treatment for PTSD [46, 47]. The presentation and the format of the material in step-2 is the same as in step-1. Expressive writing (structured written assignments) is used as an alternative to imagery exposure [48]. iCBT Step-2 is more individualised than iCBT step-1 with weekly therapeutic support via mail. There is no partner content in this step since the empirical evidence is based on an individual treatment protocol (Table 2).

**Table 2:** Overview of the of the iCBT step-2

Module	Content
1	Introduction to treatment, psychoeducation
2	Identify & recognize symptoms, breathing retraining (continued through treatment)
3	In vivo-exposure (continued through treatment)
4	Refined in vivo-exposure + intro to expressive writing
5	Expressive writing, imaginal exposure
6	Refined imaginal exposure
7	Finding hot-spots, recovery
8	Summary, maintaining progress, relapse prevention

Note. Every module contains homework assignments based on the content of the module

#### Tau

In all our four study-centres the women rate their overall childbirth experience on a Likert-scale before leaving the hospital. Women who rate their childbirth experience ≤5 are contacted 8 weeks postpartum. All participants in the study are treated according to international guidelines by the local health care system regardless of treatment allocation. TAU includes conventional support according to existing practice at the Department of Obstetrics and Gynaecology in the participating hospitals. TAU can include an opportunity for a follow-up visit with the physician and/or counselling in a fear of birth-unit (often called Aurora clinic) where specially trained midwives and obstetricians work together to help the woman deal with negative experiences from childbirth and with fear of childbirth. Although guidelines exist, the nature of counselling offered differs among hospitals in Sweden [49]. However, the intention in all four study centres is to offer post-partum counselling if needed before the women leave the hospital.

Participants are not prohibited or discouraged from seeking and receiving other care during the study period. If participants signal psychological problems (via email, telephone or questionnaires) they will be contacted and referred to appropriate care.

### Data collection

Participants log in and respond to the questionnaires via the U-CARE web portal at seven time points. The original data are electronically stored on the U-CARE web portal and can only be retrieved by members of the research group. Medical and obstetric data related to the birth will be collected from the patients’ records. A data monitoring committee is not employed; however, suicidal ideation is monitored by the psychologist (see ethical considerations below). The principal investigator (ASS) of the study will restrict access to the final trial data set.

The inclusion of participants for this study started in September 2013. The last day for recruitment of eligible was on the 31st of January 2018 and the last participant was randomised on the 12th of April 2018.

### Measurements

Overall childbirth experience is a paper and pencil administered assessment. The information and instruction are as follows; “We would like to know how you experienced your childbirth. Circle the number between 0 – 10 that best correspond to the overall experience of your childbirth. 0 corresponds to ‘A very bad experience’ and 10 corresponds to ‘A very good experience.’” A low rating, ≤ 5, indicate a negative childbirth experience. For an overview of timing of measurements and data collection please see Table 3.

**Table 3:** Timing of measurements and data collection during the JUNO study

	Prenatal	Postnatal	Baseline	Post, 6 weeks	Post, 14 weeks	1 year FU	2 years FU	3 years FU	4 years FU
The mother									
The Medical Birth Register	x	x							
The National Patient Register			x	x	x	x	x	x	x
The Swedish Prescribed Drug Register			x	x	x	x	x	x	x
Overall childbirth experience		x							
Demographics			x						
TES, EPDS^a^			x	x	x	x	x	x	x
SWLS, WCQ, ENRICH, R-DAS, PBQ, EQ-5D, SF-36			x	x	x	x	x	x	x
The partner									
WCQ, PBQ, SWLS, R-DAS, ENRICH, EQ-5D, SF-36			x	x	x	x	x	x	x

Note. Participants are randomized after completion of baseline measures

*TES* The Traumatic Event Scale, *EPDS* Edinburgh Postnatal Depression Scale, *SWLS* The Satisfaction with Life Scale, *WCQ* The Ways of coping questionnaire, *ENRICH* The communication subscale from the Evaluation and Nurturing Relationship Issues, Communication and Happiness, *R-DAS* The Revised Dyadic Adjustment Scale, *PBQ* The Postpartum Bonding Questionnaire, *EQ-5D* EuroQol 5D, *SF-36* Short Form Health Survey–36, *HADS* Hospital Anxiety and Depression Scale

^a^ Primary outcome measures

### Demographics and health history

Demographic data including information about age, level of education, ethnicity and marital status are collected at baseline. Information about previous pregnancies and births are also collected at baseline. Obstetric data regarding pregnancy, childbirth, the postpartum period and other medical data concerning the current childbirth are collected from the patients’ records (see below).

### Primary outcome measures

*The Traumatic Event Scale (TES)* measures post-traumatic stress symptoms related to childbirth, 3 months after delivery [50]. The scale was developed to assess the DSM-IV [8] criteria for PTSD FC. It comprises the stressor criterion A, with the four items formulated as follows; 1) The childbirth was a trying experience, 2) The childbirth was a threat to my physical integrity, 3) During the childbirth I was afraid that I was going to die, 4) During the childbirth I felt anxious/helpless/horrified. The response options for the A criterion are “not at all, somewhat, much, very much”, The A criterion is met if the alternatives “much” or “very much” is chosen on item 1, 2 or 3, and 4. The three categories of PTSD symptoms (in total 17 items) assess re-experiencing (B-criterion), avoidance (C-criterion), and arousal symptoms (D-criterion). The response options for these three categories are “never/ not at all, rarely, sometimes, often” and to be accounted for as presence of a symptom, the alternatives “sometimes” or “often” are required. In accordance with the DSM-IV one of five B items, three of seven C items, and two of five C items are required to fulfil the criteria respectively. The duration criterion (E) is fulfilled if symptoms have been present for 1 month at least. The influence criterion (F) is met if the degree to which it influenced life was ≥ 6 with regard to at least one of the symptoms. The 17 symptom criteria are summed and higher score indicate higher prevalence of symptoms. The symptom scores have shown good internal reliability (*α* = .84) and split half reliability (.90). The difference between the two study arms as concerns the proportion of participants classified as fulfilling the criteria for PTSD at all assessment points will be reported. Means for the summed symptoms score (criteria B, C, and D) will be compared between the study arms. The TES has been adapted for this particular traumatic event (childbirth) where questions point directly at the childbirth [50]. The scale has been developed and evaluated in a Swedish context and thus is considered clinically relevant.

*Edinburgh Postnatal Depression Scale (EPDS)* [51] measures symptoms of depression. It consists of 10 items, rated from 0 to 3, where higher summed scores indicate more symptoms of depression. The summed score ranges between 0 and 30 where a higher score indicate more symptoms. A cut-off point of ≥12 for postpartum depression is often used for screening in clinical settings [52] and is used in this study as well. A population-based study on the Swedish version showed good validity in comparison with a clinical interview [52]. The EPDS is a widely used screening measure of post-natal depression with a sensitivity of 86%, specificity of 78%, high standardised Cronbach’s *α* (.87) and split-half reliability (.88) [51]. Between the two study arms, the difference in the proportion of participants with postpartum depression according to cut-off and means for the summed scores at all assessment points will be reported. EPDS is the most frequently used instrument to screen for post-natal depression and its use will enable comparisons between studies.

### Secondary outcome measures

*The Satisfaction with Life Scale (SWLS)* measures quality of life and well-being [53]. The five items are answered on a 7-point Likert-type scale, it ranges between 5 and 35, and higher scores indicate higher satisfaction with life. A summed score will be used in this study. The scale has shown good convergent validity, good internal consistency and test-retest correlation (*α* = .87 and *r* = .82 respectively) and a single factor solution replicated through factor analysis [53, 54].

*The Ways of coping questionnaire (WCQ)* is a self-report questionnaire designed to assess and identify thoughts and actions individuals use to cope with a stressful event [55]. A version with five of the eight subscales was used in this study; confrontive coping, self-controlling, seeking social support, escape-avoidance, and planful problem solving. It consists of 33 items rated on a 4-point Likert scale (0–3) and each subscale will be summed for a raw sum score. Higher scores indicate higher use of the coping method. According to the manual the internal consistency is acceptable (α .61–.79).

*The communication subscale from the Evaluation and Nurturing Relationship Issues, Communication and Happiness (ENRICH)* assesses feelings and attitudes towards communication in the relationship [56]. It consists of 10 items responded to on a 5-point Likert scale (range 10–50) and the summed score will be used in this study. Higher scores indicate more positive feelings and attitudes toward the partner. Discriminative and concurrent validity has been established [57]. The subscale showed good test-retest and internal consistency in a Swedish sample (.77 and .89 respectively) [56].

*The Revised Dyadic Adjustment Scale (RDAS)* assesses relationship quality [58]. The RDAS consists of 14 items designed to measure adjustment in dyadic relationships on three subscales: Consensus, Satisfaction, and Cohesion. Higher scores indicate higher consensus, satisfaction and cohesion. A total summed score will be used in this study ranging between 0 and 69. In past research, the RDAS has demonstrated support for construct validity, good internal consistency (.80 to .85 and .90) respectively for the subscales and the total score as well as good split-half reliability [58]. Cronbach’s alphas from three university clinics were .86, .87, and .89 while the test–retest correlation was .82 [59].

*The Postpartum Bonding Questionnaire (PBQ)* is a screening instrument for mother–infant bonding disorders and it is designed to provide an early indication of disorders within mother-infant bonding, a high score indicates more pathological responses [60]. The four sub-scales (Impaired bonding, Rejection and Anger, Anxiety about care and Risk of abuse) each provide a summed score from the responses on the six-point Likert-scale (0 to 5) and the total score ranges from 0 to 125. This study will report subscales and total score. The four sub-scales showed moderate sensitivity (1.0, 0.89, 0.56 and 0.28, respectively) compared to the Birmingham Interview for Maternal Mental Health and high specificity (0.85, 1.0, 0.96 and 1.0, respectively) for mother–infant bonding disorders. Test–retest reliability was .95, .95, .93 and .77 for the four sub-scales [60, 61].

*EuroQol 5D (EQ-5D)* instrument will be used to estimate the quality adjusted life years (QALYs). It is one of the most widely used health utility measures [62]. The EQ-5D is a generic instrument of health-related quality of life. It records self-reported problems in each of the five domains: mobility, self-care, usual activities, pain/discomfort and anxiety/depression. Each domain is scored/rated based on three levels of severity corresponding to no problems, some problems, and extreme problems. This enables one to obtain a population-based preference score or societal index (SI). The EQ5D-3 L tool also has a visual analogue scale (VAS) that asks participants to rate their current health from 0 (worst imaginable health) to 100 (best imaginable health) [63]. Both the index scores and the VAS score correlated highly with measures of quality of life, anxiety and depression as well as responsive to change in patients with anxiety disorders [64]. Swedish norms are available for the five domains and the VAS scale, by gender, and different age groups, as well as for the population [65].

*Short Form Health Survey – 36 (SF-36)* is a self-administered, short-form health survey with 36 questions. It yields an 8-scale profile of functional health and well-being scores. The SF-36 is composed by eight subscales, each of which range from 0 to 100. A high score indicates better health (range 0–100) [66]. A total score of 50 corresponds to the mean of the general population with a standard deviation of 10. The SF-36 has demonstrated good internal consistency and factorial validity [67]. It will be used to capture the HRQoL of the study participants. This will be used in the health economic evaluation analysis (cost utility analysis). The SF-36 tool was chosen because it is sensitive and relatively well correlated with mental health disease specific tools e.g. DASS-21 for Depression [68].

### Outcomes for the partner

The partner answers WCQ, PBQ, SWLS, R-DAS, the communication subscale from ENRICH, EQ5D, and the SF-36 (see above). In addition, the Hospital Anxiety and Depression Scale (HADS) [69] is used to assess symptoms of anxiety and depression among the partners. It was developed to assess anxiety and depression symptoms among somatic care seeking patients during the previous week. There are seven items each for anxiety and depression. The responses are rated on a 4-point Likert scale and the scores are summed within depression and anxiety. The scores range between 0 and 3 for both depression and anxiety resulting in a range of 0 to 21 for each scale. Higher scores indicate more symptoms. In a review of 747 papers it was concluded that the HADS shows good validity and reliability scores [70]. Studies have reported good validity and reliability, with good internal consistency for anxiety (α = .84) and depression (α = .82) as well as for the full scale (α = .90) among Swedish respondents [71].

### Swedish national registers

#### The medical birth register

Information from the prenatal care; data on the mother, smoking and use of oral tobacco, cohabitation status, information on previous pregnancies, maternal medical drug use during pregnancy, diagnoses before and during pregnancy. Information from the delivery care; maternal diagnoses, mode of delivery, foetal presentation, analgesia and anaesthesia and hospital code. Information from the neonatal care: the infants birth weight, body length and head circumference, single or multiple birth, duration of pregnancy, Apgar score, infant diagnoses, live birth and/or stillbirth.

#### The National Patient Register

Patient data; personal registration number, gender, age, place of residence. Geographical data; county, hospital/clinic, department. Administrative data on in- and outpatients; Date of admission and discharge, length of stay, unplanned/planned admission, admitted from and discharged to. Acute care data; type of department, date and time point (admission, assessment by physician, discharge, terminated visit). Compulsory admission and detention under the Swedish Mental Health Act and Forensic Psychiatric Care. Medical data; main and secondary diagnosis, external cause of injury and poisoning, and procedures.

#### The Swedish prescribed drug register

Dispensed item (e.g. substance, brand name, dosage, amount), prescription (e.g. date of the prescription, and the date when the goods are taken out), costs (county cost and customs fee) characteristics of the workplace where the prescription was made and the profession of the prescribers.

### Health economic evaluation

A health economic evaluation in the form of a cost utility analysis will be conducted. It will focus on assessing whether iCBT + TAU is good value for the money spent compared to TAU in the management of PTSD FC and depression symptoms. The analysis will take a societal perspective as recommended by the resource allocation agencies in Sweden. The cost utility evaluation will be conducted alongside the JUNO-trial and thus intervention costs will be derived/collected from the trial data. The resource use data will be mainly collected from registers (see above), as the most important parameters here are healthcare usage and productivity losses by the mothers. The outcome measure for the study will be Health-related Quality of Life (HRQoL) captured using the EQ5D-3 L tool/SF-36 and translated into utilities. Uncertainty in the work will be addressed through sensitivity analysis and the work will be presented to a subgroup analysis level.

Reason for drop out (for mothers and their partner) will be investigated 1 year after informed consent was returned; The following open-ended question will be addressed to those who were randomized to the iCBT condition but dropped out and to those who consented to participate but did not log in to the UCARE portal; “Some time ago you were invited to, and consented to participate in, the JUNO study. What was the reason for you to not participate?”

### Statistical analysis

Data will be imported from the U-CARE web portal. For demographic data, χ^2^ and t-test will be used to examine differences between the groups at baseline. For the continuous data, a mixed model repeated measure (MMRM) will be used to evaluate treatment effects. All available data from all participants are used so this will be a suitable approach for intent-to-treat analysis. Effect sizes (Cohen’s *d*) will be based on suggestions for repeated measures and multi-level designs [72, 73] and effect sizes for planned contrasts for interactions [74]. For the binary outcome data *χ*^2^ analysis and calculations of odds-ratios will be performed. Per-protocol (defined as a minimum of four completed weeks out of six in step-1 and a minimum of six completed weeks out of eight in Step-2), completer, and drop-out analysis will be performed. Exploratory sub-group analyses will be performed between those with negative birth experience, immediate caesarean section and those with a major postpartum haemorrhage. We will also investigate differences between primiparas and multiparas, those with/without vacuum extraction, those with/ without partner involvement, as well as those with/without pro-longed labour. We will also consider known risk factors for PTSD FC as covariates.

During the study period, data from all mothers giving birth at the four trial sites will be collected from their patient records. In order to describe and compare our sample with this population we will collect a) obstetric data, b) an additional rating of overall childbirth experience and c) EPDS. The additional rating of overall childbirth experience and the EPDS is assessed at the first visit to maternal care after the birth (approximately 6–8 weeks). Subgroup investigations will be performed among those who; declined participation but were eligible, did not respond to the invitation but were eligible, were eligible but not invited (missed) and all who rated overall childbirth experience. Finally, we will report reasons for not participating among those who consented but did not participate. Statistical analyses will be performed by SPSS (IBM SPSS Statistics).

### Ethical considerations

The Regional Ethics Review Board in Uppsala has approved the trial (2012/495/1). The U-CARE portal is used for data collection and delivery of interventions. A review of the literature suggest that it is not likely that any adverse effects will result from internet-based support activities. Rather, there is good reason to assume that various types of internet-based support and CBT may be effective in treating mental problems. The primary ethical risk is enrolment of participants in need of other specialized medical interventions, such as those with severe depression. Those patients will be referred to psychiatric care. Careful baseline measurement prior to inclusion in the study is designed to minimize that risk. Far-reaching measures have been taken to ensure that the carebase.se IT platform will be secure against unauthorized access or intrusion, and that unauthorized persons are unable to link the personal data of study participants with patient-reported data. Study participants may at any time terminate participation without giving any reason for doing so. Research subjects in the intervention group may benefit from the project if iCBT leads to decreased anxiety and depressive symptoms and a better health-related quality of life compared with conventional care. Patients reporting suicidal ideation will be excluded from the study and be contacted by the psychologist for assessment and (if needed) referred to other treatment. Likewise, if there is any reason to believe that a participant has become suicidal during the study, the psychologist will contact the patient and if needed refer the patient to a professional who can offer appropriate treatment. Those who suffer harm from trial participation are insured via the Patient Injury Act.

Any modifications to the protocol of the study that may impact the participants will lead to a formal addendum to the Regional Ethics Review Board for their approval. These modifications will be communicated in forthcoming publications and added in the trial registry (ISRCTN).

Consent for participation is mailed to the principal investigator. Participants’ confidentiality is protected during the whole study period. Written consent forms and any personal information are stored separately. Personal information is accessed through participants’ records at the trial sites’ hospitals and through the web portal. Access to these data is restricted by the Swedish health care law and approval from the principal investigator respectively.

### Monitoring

The research team will conduct monitoring of trial conduct. Recurrent visits to each trial site will be made by JS and a research assistant. Adherence to enrolment protocol and invitations to study participation will be audited and if necessary corrective actions will be promoted. Furthermore, MJ, ASS and JS will have recurrent contact with key personnel at the trial sites on a (3 month) schedule to monitor enrolment and invitation of participants. In addition, a reference group (advisory board) will meet twice a year to overlook and give advice on the status of the trial and on any unforeseen events. The reference group consists of the authors of this publication and expertise in obstetrics and gynaecology; senior professor Ulf Högberg (MD, PhD), professor Inger Sundström Poromaa (MD, PhD), professor Alkistis Skalkidou (MD, PhD), Frida Viirman (registered midwife and registered nurse), and professor Gunilla Sydsjö (PhD, registered psychotherapist). Associate professor Inna Feldman (PhD, health economist) was consulted for the health economic analyses.

## Discussion

Most women with negative experiences and PTSD FC have the ability to handle the psychological stress it may cause, but in previous studies a significant proportion of women have reported symptoms of posttraumatic stress, anxiety and depressive symptoms that need attention. However, little is known about how to treat psychological reactions in women with negative experiences from birth and it is unclear how symptoms are perceived and how they develop over time. For these reasons there is a growing recognition of the need to offer interventions for those suffering from mental illness related to reproductive events, and at the same time there is almost no published research on treatment for PTSD FC. To the best of our knowledge, only one RCT has examined the effect of iCBT for women with PTSD [42]. This study showed good outcome at the end of treatment for iCBT compared with a wait list condition. It is however uncertain whether trauma focused treatment for PTSD caused by events other than childbirth are suitable for those with negative experiences FC, and whether the long-term effects are beneficial for those with PTSD FC.

It is of particular importance to consider the recruitment process in clinical trials. In our study, the majority of the women in the participating study-centres rate their overall childbirth experience as part of the standard protocol at these centres. We contact all women with a low rating and believe that we address a majority of the women with a negative experience FC who have a potential need for psychological follow up. In our study we offer minimal therapeutic support which may affect compliance from the participants. We also know that people with trauma-related psychological problems often display avoidant behaviour and therefore some women may be reluctant and not motivated to accept offered psychological treatment.

Findings in this study will contribute knowledge and understanding of women with negative birth experiences and PTSD FC. The study will yield information about the psychological status in this group and a better understanding of potential cost-effective interventions that could improve women’s psychological health and quality of life. Hopefully this might reduce health care costs and promote positive bonding/relationship to the child and partner.

From a public health perspective, it is important to address and prevent the development of mental illness among women of childbearing age. Adverse mental health after childbirth might have life-long consequences for the individual, couple and family and would have considerable implications for public health. Developing effective internet-based care could have a major public health impact with respect to improvements in accessibility and cost-effectiveness. ICBT has advantages over traditional face to face CBT; the patient can work with the material at any time preferred and in her own pace [75] which might be suitable for new mothers. By offering support via the internet it is possible to reach a larger population. Using the internet can also be a cost-effective way to administer care and support since the therapist can treat more patients in less time [76]. For proper priority setting and decision making, it is therefore essential with health economy evaluations.

Clinical recruitment addresses a broader part of the target population, while recruitment via media has been criticized because it may result in biased samples and thus result in studies of limited generalizability. Potential weaknesses in this study could be the risk of missing a few women who develop symptoms later in the process, and to over-include women who have a reaction close to birth but who will return to normal well-being quickly. Another limitation is that we do not screen for earlier psychiatric conditions, therefore we cannot be sure whether a particular woman’s estimation of mental illness is linked to the birth exclusively and/or also previous psychiatric symptoms. The results of the trial will be disseminated regardless of the magnitude or direction of effect as described in this study protocol. There is no publication restriction attached to this study. The primary publications from this trial focus on outcome effects. Ancillary publications using the collected data as described in this study protocol will be decided on by the reference group. Authorship eligibility will be discussed in this group as well and a professional translator will be consulted prior to submission of any reports.

## Abbreviations

EMDR: Eye movement desensitisation reprocessing
ENRICH: The communication subscale from the evaluation and nurturing relationship issues, communication and happiness
EPDS: Edinburgh Postnatal Depression Scale
EQ-5D: EuroQol 5D
ES: Effect size
HADS: Hospital anxiety and depression scale
Health history: Information about previous pregnancies and births are collected at baseline
HRQoL: Health-related quality of life
iCBT: Internet delivered cognitive behaviour therapy
MMRM: Mixed model repeated measure
Obstetric data regarding pregnancy: Childbirth, the post partum period and other medical data concerning the current childbirth
PBQ: The Postpartum Bonding Questionnaire
PTSD FC: Posttraumatic stress disorder following childbirth
RCT: Randomised controlled trial
RDAS: The revised dyadic adjustment scale
SF-36: Short Form Health Survey – 36
SWLS: The satisfaction with life scale
TAU: Treatment as usual
TES: The traumatic event scale
U-CARE: Uppsala University Psychosocial Care Programme
WCQ: The Ways of coping questionnaire
